# Physical training reverses changes in hepatic mitochondrial diameter of Alloxan-induced diabetic rats

**DOI:** 10.1590/S1679-45082018AO4353

**Published:** 2018-08-06

**Authors:** Gabriel Keine Kuga, Rafael Calais Gaspar, Vitor Rosetto Muñoz, Susana Castelo Branco Ramos Nakandakari, Leonardo Breda, Bruna Marina Sandoval, Flávio Henrique Caetano, José Alexandre Curiacos de Almeida Leme, José Rodrigo Pauli, Ricardo José Gomes

**Affiliations:** 1 Universidade Estadual Paulista “Júlio de Mesquita Filho”, Rio Claro, SP, Brazil.; 2 Faculdade de Ciências Aplicadas, Universidade Estadual de Campinas, Limeira, SP, Brazil.; 3 Fundação Hermínio Ometto, Araras, SP, Brazil.; 4 Universidade Paulista, São Paulo, SP, Brazil.; 5 Universidade Federal de São Paulo, Santos, SP, Brazil.

**Keywords:** Diabetes mellitus, Exercise, Liver, Wistar, rats, Diabetes mellitus, Exercício, Fígado, Ratos Wistar

## Abstract

**Objective:**

To investigate the effects of physical training on metabolic and morphological parameters of diabetic rats.

**Methods:**

Wistar rats were randomized into four groups: sedentary control, trained control, sedentary diabetic and trained diabetic. *Diabetes mellitus* was induced by Alloxan (35mg/kg) administration for sedentary diabetic and Trained Diabetic Groups. The exercise protocol consisted of swimming with a load of 2.5% of body weight for 60 minutes per day (5 days per week) for the trained control and Trained Diabetic Groups, during 6 weeks. At the end of the experiment, the rats were sacrificed and blood was collected for determinations of serum glucose, insulin, albumin and total protein. Liver samples were extracted for measurements of glycogen, protein, DNA and mitochondrial diameter determination.

**Results:**

The sedentary diabetic animals presented decreased body weight, blood insulin, and hepatic glycogen, as well as increased glycemia and mitochondrial diameter. The physical training protocol in diabetic animals was efficient to recovery body weight and liver glycogen, and to decrease the hepatic mitochondrial diameter.

**Conclusion:**

Physical training ameliorated hepatic metabolism and promoted important morphologic adaptations as mitochondrial diameter in liver of the diabetic rats.

## RESUMO

**Objetivo:**

Investigar os efeitos do treinamento físico nos parâmetros morfológicos e metabólicos de ratos diabéticos.

**Métodos:**

Ratos Wistar foram randomizados para quatro grupos: controle sedentário, controle treinado, diabético sedentário e diabético treinado. *Diabetes mellitus* foi induzido por administração de Aloxana (35mg/kg) nos Grupos Diabético Sedentário e diabético treinado. O protocolo de treinamento físico incluiu natação com carga de 2,5% do peso corporal, por 60 minutos por dia (5 dias por semana) para os Grupos Controle Treinado e diabético treinado, durante 6 semanas. Ao final do experimento, os ratos foram sacrificados, e o sangue foi coletado para determinação das concentrações séricas de glicose, insulina, albumina e proteínas totais. Amostras do fígado foram coletadas para determinação do glicogênio, proteínas, DNA e diâmetro mitocondrial.

**Resultados:**

O Grupo Sedentário Diabético apresentou redução no peso corporal, insulinemia e glicogênio hepático, além de maior glicemia e diâmetro mitocondrial hepático. O protocolo de treinamento físico em animais diabéticos foi eficiente para restaurar o peso corporal e o glicogênio hepático, além de reduzir o diâmetro mitocondrial hepático.

**Conclusão:**

O treinamento físico melhorou o metabolismo hepático e promoveu importantes adaptações morfológicas, como no diâmetro mitocondrial no fígado de animais diabéticos.

## INTRODUCTION

*Diabetes mellitus* (DM) is a disease characterized by inefficient secretion or action of insulin, classified into two main types, 1 and 2. Type 1 *diabetes mellitus* (insulin-dependent) is related to insulin deficiency due to mechanisms that reduce serum concentration of this hormone. On the other hand, type 2 DM (non-insulin-dependent) is characterized by insulin resistance, a condition when this hormone does not act correctly, even if its serum concentration is high.^1^

Insulin is a polypeptide anabolic hormone that plays an important role in protein, carbohydrate and lipid metabolism. In cases of deficiency of this hormone, or its ineffective action, glucose and amino acid uptake is impaired, resulting in hyperglycemia due to increased gluconeogenesis, lipolysis and degeneration in several tissues, primarily renal, cardiovascular, bone and hepatic.^1,2^ In the liver, DM reduces glycogen storage, alters secretion of several hormones, such as insulin-like growth factor (IGF), and induces morphological alterations.^3-5^ Moreover, when there is no appropriate metabolic control, DM triggers hepatic steatosis due to lipid accumulation in the liver.^5,6^

On the other hand, physical exercise is an essential component of DM treatment (types 1 and 2). Thys therapy increases several metabolic parameters, such as muscular glycogen during activity, lipid contribution to energy metabolism, number and size of muscular mitochondria, insulin sensitivity and important enzymes responsible for lipid mobilization and oxidation.^7-9^ In addition, physical exercise contributes to peripheral amino acid and glucose uptake, helps preserve muscular glycogen storage, and reduces protein catabolism typical of type 1 diabetes.^2,10,11^

Alloxan is a widely used drug to induce 1 DM type in experimental models, for destroying the pancreatic beta cells, causing alterations in glycemic homeostasis in the animals (*e.g.*, hyperglycemia, reduction of hepatic glycogen and reduced circulating insulin).^3,5,12,13^ Furthermore, the induction of DM by Alloxan provides an experimental background for investigating the effects of physical training as a therapeutic intervention for this disease.^3,5,12,13^

## OBJECTIVE

To investigate the effects of a physical exercise protocol on the metabolic and ultrastructural aspects of hepatic tissue of experimental diabetic animals.

## METHODS

### Experimental animals

Male Wistar rats (70-day old) were used in the experiments. The animals were housed in a room at 25°C, on a 12-hour light/dark cycle and received Purina rat chow and water *ad libitum*. *Diabetes mellitus* was induced by an intravenous injection of monohydrate Alloxan (35mg.kg^-1^ body weight; Sigma^®^).^4,12,14^ After 5 days, blood samples were obtained from rats who had been fed to determine plasma glucose concentration. Rats that were not diabetic (glucose <14.7mmolL^-1^) or too severely diabetic (glucose >35.5mmolL^-1^) were excluded from the study.^12^ This procedure was in accordance with the *Comitê de Ética em Pesquisa com Animais* of the *Universidade de Campinas* (Process 4513-1/2017). For the experiment, the rats were randomly allocated into the groups (n=10 per group): Sedentary Control (SC), Trained Control (TC), Sedentary Diabetic (SD) and Trained Diabetic (TD). The number of animals utilized for the analysis is represented in the legend of the figures.

### Physical exercise protocol

The exercise protocol consisted of swimming during 60 minutes per day, 5 days a week, during 6 consecutive weeks. After an adaptation period of 5 days to aquatic environment, we utilized loads of 2.5% body weight attached to animal chest. All swimming sessions started at 8 a.m. and were performed in a tank (100cm x 70cm x 60cm), with water at 31°C±1°C, 40cm deep.

### Euthanasia and blood biochemical profile

At the end of experimental period, all rats rested for 48 hours after the last bout of exercise, with fasting. After euthanasia, blood samples were collected in glass tubes with no anticoagulant to evaluate various parameters. All blood samples were centrifuged at 3,000rpm, for 10 minutes, and the following analysis were performed in the serum samples: glucose by colorimetric enzymatic method,^15^ insulinemia (radioimmunoassay, kit Coat-A-Count, Diagnostic Products Corporation, Los Angeles, CA, United States), and serum total protein and albumin.^16^

Laparatomy was performed to extract liver fragments and measure glycogen, total protein and DNA. The following protocols were performed: glycogen content was obtained following the method described by Dubois et al.,^17^ total protein content by the method of Lowry et al.,^18^ and DNA as per Giles et al.,^19^ We also evaluated the protein/DNA ratio to measure the protein metabolism in the liver.

### Routine for electronic microscopy

Fragments of left hepatic lobe (three animals per group) were fixed in modified Karnovisky for 3 hours, and later placed in 5mL of glutaraldehyde 50% and 95mL of phosphate buffer 0.1M. The sample treatment for this procedure was previously described^4^ and adapted from Reynolds method.^20^ The samples were analyzed and photographed in the transmission electron microscope Philips CM10. The mitochondrial diameter was measured utilizing the AUTOCAD^®^ software.

### Statistical analysis

All results were expressed as mean±standard deviation and analyzed using analysis of variance (ANOVA), with Bonferroni post-hoc test. The significance level was established in 5%.

## RESULTS

Table 1 shows the evolution of body weight in all groups. After the fourth week of experimental period, the SD Group exhibited lower body weight than SC. By the end of the physical training, the TC Group also showed higher body weight than SD. This data indicates that DM induced weight loss.

**Table 1:** Evolution of body weight of rats in all groups during experimental period (6 weeks)

Parameter	Groups	Week 1	Week 1	Week 3	Week 4	Week 5	Week 6
Body weight (g)	SC	298±30	322±41	324±53	351±29	364±69	379±55
	TC	290±32	305±55	316±55	316±33	337±55	352±43
	SD	256±38	264±47	274±47	280±61*	275±65*	277±49*^†^
	TD	250±39	290±32	301±56	309±54	316±58	327±54

Results expressed as mean±standard deviation. * p<0.05 *versus*

SC Group; ^†^ p<0.05 *versus* TC Group. SC: Sedentary Control; TC: Trained Control; SD: Sedentary Diabetic; TD: Trained Diabetic.

We evaluated whether DM and physical training modulated serum parameters of the animals. Diabetic animals showed higher levels of glucose and lower levels of insulin, confirming the pathological state. There were no differences between the Control Groups or as consequence of physical training for these parameters (glucose and insulin). All groups exhibited similar levels in relation to serum total protein and albumin levels (Table 2).

**Table 2:** Serum parameters of rats in all groups during experimental period (6 weeks)

Serum parameters	SC	TC	SD	TD
Glucose, mg/dL	117±13	121±15	439±99*^†^	413±91*^†^
Insulin, mIU/mL	15.2±2.9	16.3±3.4	11.8±2.1*^†^	11.5±2.9*^†^
Total protein, g/100 mL	6.58±0.53	6.36±0.36	6.48±0.72	6.34±0.39
Albumin, g/100 mL	4.63±0.93	4.39±0.8	4.21±0.8	4.32±1

Results expressed as mean±standard deviation. * p<0.05 *versus*

SC Group; ^†^ p<0.05 *versus* TC Group. SC: Sedentary Control; TC: Trained Control; SD: Sedentary Diabetic; TD: Trained Diabetic.

We also analyzed hepatic parameters (Table 3). First, the SD Group showed lower content of glycogen than the other groups, confirming the catabolic state of diabetic animals. Later, we did not find differences between the groups for total protein and DNA. However, the TC Group showed higher protein/DNA ratio than SC.

**Table 3:** Hepatic parameters of rats in all groups during experimental period (6 weeks)

Hepatic parameters	SC	TC	SD	TD
Glycogen, mg%	5.2±1.2	6.2±1	2±0.5*^†‡^	5±1.8
Total protein, mg%	2.2±0.38	2.8±0.62	3±0.77	3±1.1
DNA, mg%	0.18±0.01	0.14±0.04	0.18±0.04	0.18±0.03
Protein/DNA	12±2.8	20±6.4*	17±3.5	17±5.9

Results expressed as mean±standard deviation. * p<0.05 *versus*

SC Group; ^†^ p<0.05 *versus* TC Group; ^‡^ p<0.05 *versus* TD. SC: Sedentary Control; TC: Trained Control; SD: Sedentary Diabetic; TD: Trained Diabetic.

**Figure 1:**
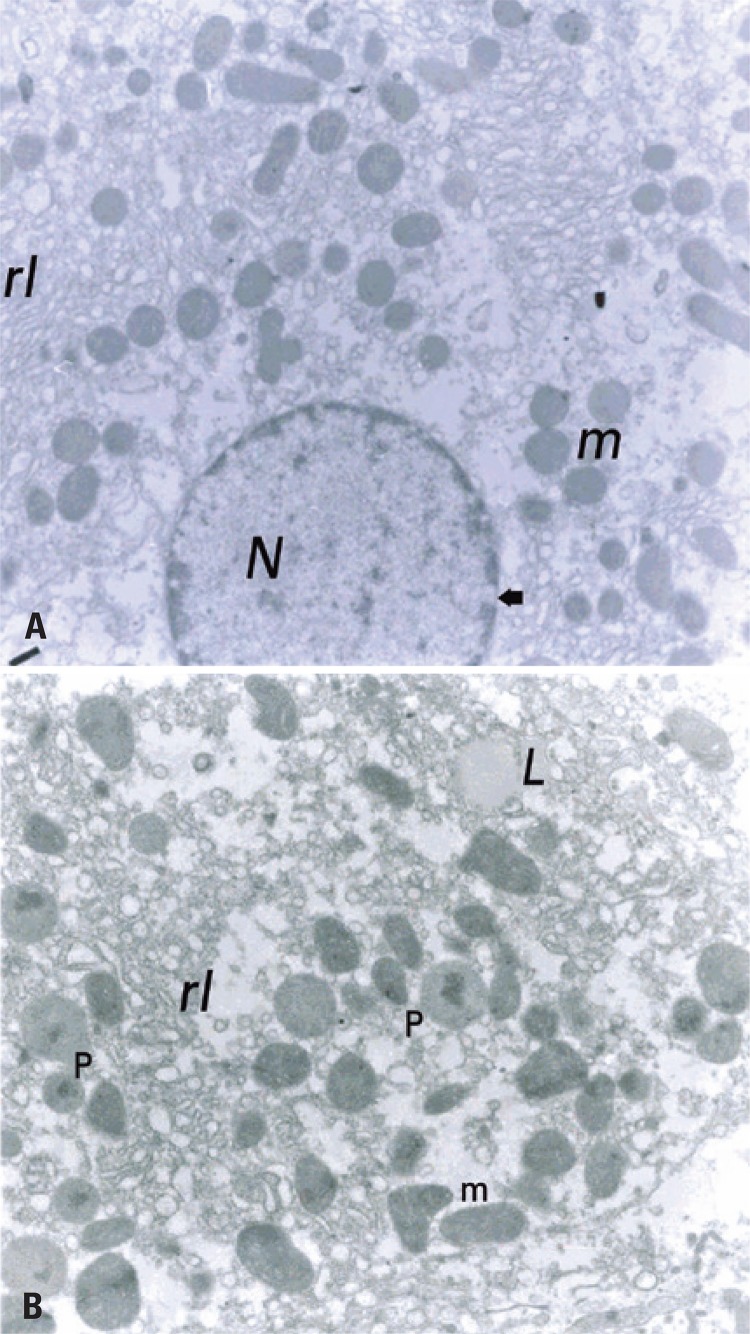
Ultrastructure of hepatic tissue of Sedentary Control Group

**Figure 2:**
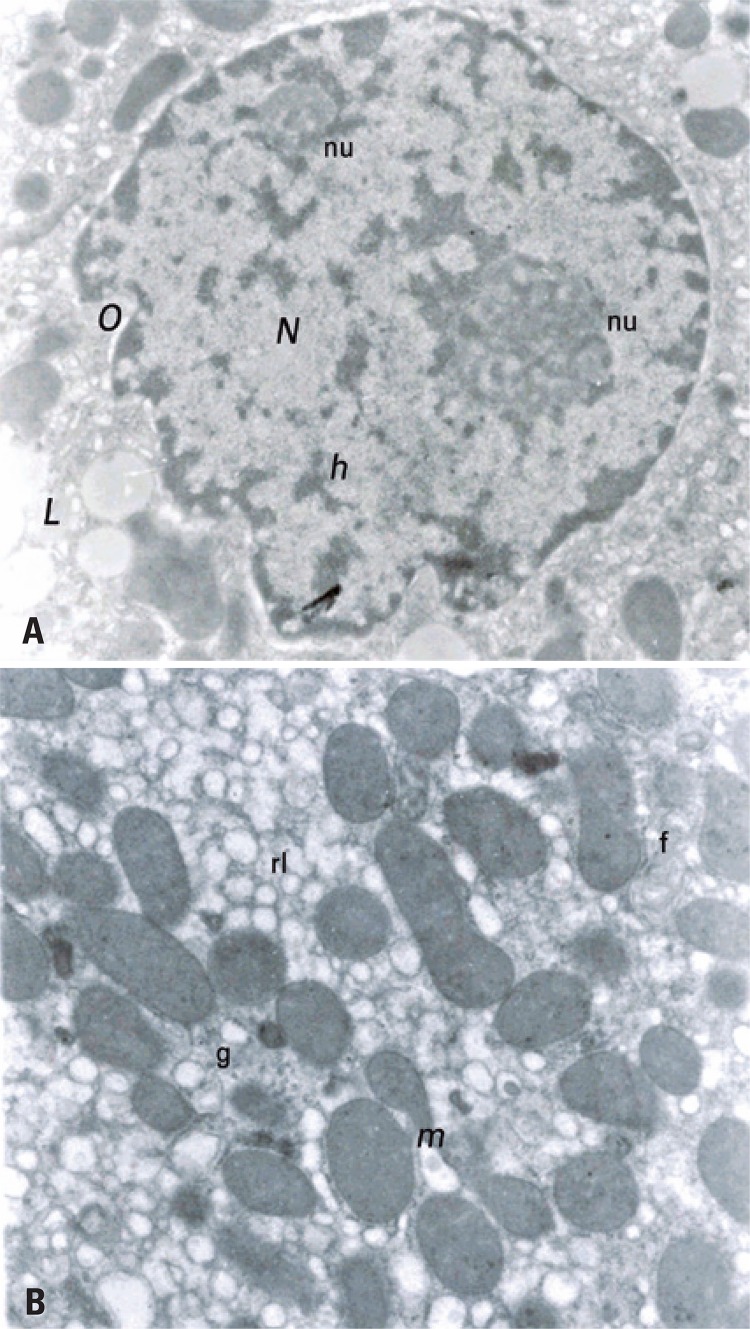
Structural characteristics of Trained Control Group

Figure 1 highlights the ultrastructure of hepatic tissue of SC Group, showing that the liver of these animals with normal structural characteristics, such as mitochondrial cristae, rough and smooth endoplasmic reticulum, peroxisomes, low lipid content, and apparently normal nuclear envelope. The TC Group had structural characteristics similar to SC, but we found a number apparently higher of peroxisomes around the mitochondria (Figure 2).

(A) Representative micrograph of rat hepatic tissue from the Sedentary Control Group. This micrograph shows the abundance of mitochondria (m) around the nucleus (N) and in the smooth endoplasmic reticulum (rl). Note some abundance of nuclear pores (arrow) (10,250x). (B) Representative micrograph of rat hepatic tissue of the Trained Control Group. This micrograph shows the abundance of peroxisomes (P) among the mitochondriae (m) and the smooth endoplasmic reticulum (rl). The presence of material with lipid aspects (L) is observed, but without the characteristic delimitation of typical droplets (14,500x).

(A) Representative micrograph of rat hepatic tissue of the Sedentary Diabetic Group. The altered appearance of the nucleus (N) and large lipid droplets (L) stand out. In addition, irregularities in the inner membrane (o) of the nuclear envelop, and the presence of smooth endoplasmic reticulum around the nucleus, as well as rough endoplasmic reticulum in smaller amounts (14,500x). (B) Representative micrograph of hepatic tissue of rat Diabetic Trained Group. The vacuolated smooth endoplasmic reticulum (rl) with is located among the mitochondriae (m). Note also the presence of glycogen (g) and myelin figures (f) (21,000x).

There were structural alterations in the SD Group, like the occurrence of ripples in the internal membrane of the nuclear envelop. In addition, the hepatocytes showed more vesiculated endoplasmic reticulum, higher amount of lipid droplets and hidden mitochondrial cristae.

Similarly to SD Group, the TD Group showed the same structural alterations, confirming the effects of DM. However, we observed an apparently larger amount of glycogen around the mitochondriae in the TD Group, suggesting an effect of physical exercise in this aspect.

**Figure 3:**
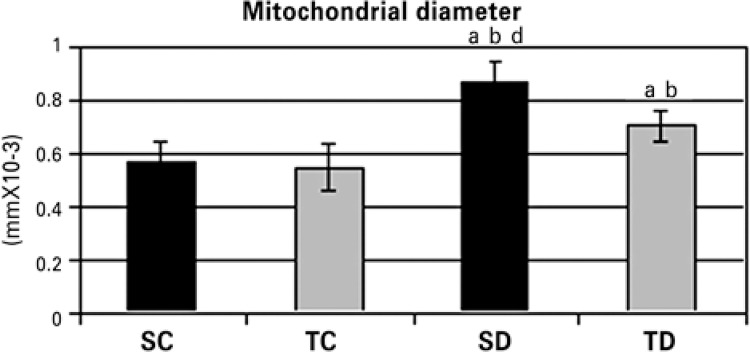
Mitochondrial diameter (mm×10-3) of rats from Sedentary Control Group (SC), Trained Control (TC), Sedentary Diabetic (SD) and Trained Diabetic (TD) after the experimental period (6 weeks; n=10 rats per group)

**Figure 4:**
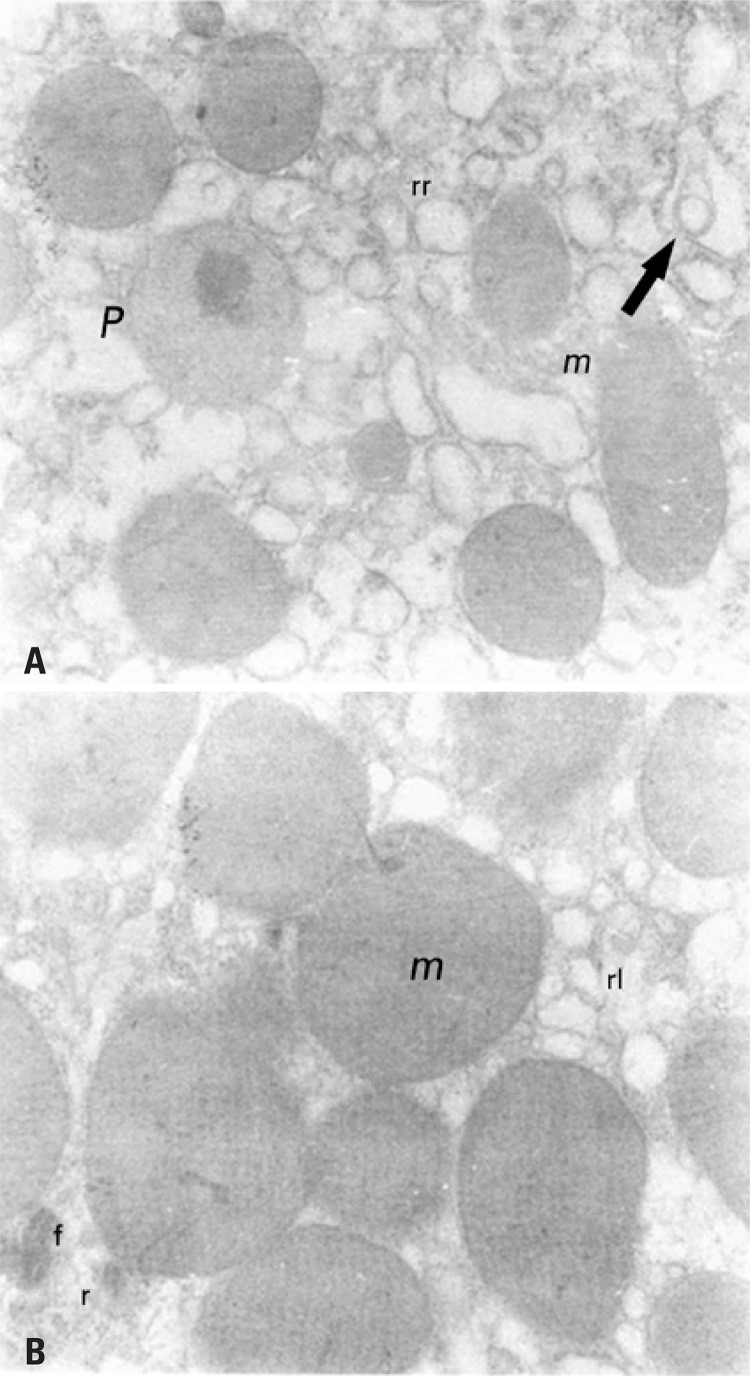
The mitochondria of Sedentary Groups

Finally, figure 3 exhibits the mitochondrial diameter of the studied groups. The statistical analysis revealed significant increase (p>0.05) of mitochondrial diameter in diabetic (SD and TD) groups as compared to Control Groups (SC and TC). The TD group had a reduction in mitochondrial diameter in comparison to SD, suggesting an effect of physical exercise. Figures 4A and 4B illustrates the mitochondrial alterations between the Sedentary Groups (SC and SD) by means of the ultrastructural technique.

Data expressed as mean±standard deviation. ^a^ p<0.05 *versus* Sedentary Control; ^b^ p<0.05 *versus* Trained Control. ^d^ p<0.05 *versus* Trained Diabetic.

(A) Representative micrograph of rat hepatic tissue Sedentary Control Group. A large amount of rough endoplasmic reticulum (rr) is observed, besides peroxosome (P) and mitochondria (m) (41,000x). (B) Representative micrograph of hepatic tissue of rat Sedentary Diabetic Group. Large vacuoles of the smooth reticulum (rl), some ribosomes (r), the presence of myelin figures and mitochondria with high diameter (m) are noted (41,000x).

## DISCUSSION

Physical exercise is an essential component of treatment for DM and the associated metabolic *deficits*. In the present study, the results obtained for the SD Group are in accordance with what is clinically expected in the DM scenario, which includes reduced weight gain, hypoinsulinemia, and hyperglycemia.^1^ This condition results from dysfunction of beta-pancreatic Langerhans cells due to administration of Alloxan.

The physical exercise protocol used does not reduce serum glucose levels of diabetic animals. There are previous studies demonstrating better response of muscle and adipose cells to insulin after physical exercise, due to increase in glucose transporters (GLUT-4) and its translocation to the membrane.^9^ After physical exercise, there are improvements in the initial steps of insulin signaling pathway, starting with increased association between the insulin receptor substrate (IRS-1) and phosphatidylinositol 3-kinase (PI3K),^7,21^ leading to better insulin sensitivity. Also, muscular contraction mobilizes the GLUT-4 in an insulin-independent pathway, contributing to higher glucose uptake.^22^ However, physical training did not have effect on serum glucose of diabetic animals in the present study, maybe due to the duration of physical training.

We did not find differences between the groups as to serum total protein and albumin, suggesting that diabetic animals did not present dehydration levels that could interfere in other results. It is well known that one consequence of DM is increase in osmotic pressure of extracellular fluids, which results in transfer of fluids causing dehydration.^2^ Hyperglycemia also causes loss of glucose through urine and osmotic diuresis, resulting in depletion of organic fluids. Diabetic proteinuria is related to increased glomerular filtration of proteins and decreased tubular reabsorption, considering that these factors are influenced by the degree of metabolic control of diabetes.^23,24^ Despite hyperglycemia, our results indicated that the level of dehydration could interfere in biochemical or morphological results.

In the present study, no significant differences were found between the groups for total proteins and liver DNA measurements. However, there was a significant increase in the protein/DNA ratio of the hepatic tissue in the TC Group, suggesting that the physical training protocol favored reduction in hepatic protein catabolism.^4^ Nevertheless, the SD Group presented reduced hepatic glycogen storage in comparison to Control Groups. This is corroborated by previous results in the literature.^10,12^ Confirming the therapeutic effect of physical exercise in diabetes, we found restored hepatic glycogen storage in TD Group as compared to SD Group. Leme et al.,^3^ also demonstrated increased hepatic glycogen in diabetic animals subjected to physical training as compared to their sedentary littermates. In our work, the physical training protocol was efficient to restore hepatic glycogen storage in diabetic animals and increase the protein/DNA ratio in the liver.

The ultrastructural analyzes of the liver revealed structural alterations promoted by DM, and increased mitochondrial diameter stood out. Our results corroborated the findings of Kozyritskiĭ et al.,^25^ who reported an increased number of mitochondriae, reduced rough endoplasmic reticulum, and proliferation of smooth endoplasmic reticulum. Additionally, bodies similar to lysosomes and autophagic vacuoles in the cytoplasm of hepatocytes in diabetic rats were observed. The smooth endoplasmic reticulum is responsible for several functions, depending on the type of cell they belong to. In hepatocytes, this organelle acts on lipid and cholesterol metabolism, contributing to detoxification process. It is also known that free or aggregated ribosomes in the cisternae of the rough endoplasmic reticulum are related to cellular protein synthesis.^26^ Petersen^27^ also found mitochondrial abnormalities in diabetic animals, suggesting that this mitochondrial hyperfunction may occur in an attempt to prevent hepatic steatosis. In the same way, Lucchesi et al.,^28^ related a decrease in the number of intracytoplasmic organelles and degeneration of mitochondria after ultrastructural analyses of hepatocytes of diabetic animals. The liver mitochondriae play a very important role in the pathogenesis of hepatic steatosis, a comorbidity quite common in diabetic patients.^29,30^

Diabetic individuals frequently present steatosis or glycogenosis. Steatosis can progress to fibrosis and cirrhosis, unlike glycogenosis that does not evolve to these pathologies, but may indicate a need for better glycemic control.^31^The accumulation of lipids in liver cells can lead to steatosis, with subsequent evolution to cirrhosis.^5^ In our study, we did not observe the occurrence of glycogenosis in hepatocytes, but we found a large accumulation of lipids in the liver of diabetic animals, a factor that may precede the onset of steatosis. We also observed changes in the chromatin mass and nuclear envelop of diabetic animals. Doi et al.,^32^ also found nuclear ripple, alterations in the chromatin mass and nucleus with irregular contour in diabetic animals. In humans, Schmid et al.,^33^demonstrated that diabetic individuals have lower synthesis of hepatic adenosine triphosphate (ATP), and this fact is related to insulin resistance, possibly presenting deficient concentration in hepatic mitochondria.^4^

In relation to TD Group, the statistical analysis showed that the mitochondrial diameter in this group was smaller than in the SD Group, suggesting that the physical training protocol used may have contributed to reduce mitochondrial hyperfunction. Few studies investigated the structural changes in the hepatic tissue of diabetic subjects and the influence of physical activity on these alterations. However, it is well known that high-fat diet and lack of physical exercise are associated with ultrastructural alterations in the liver and the prevalence of hepatic steatosis.^34^ Moreover, Lima et al.,^35^ verified that chronic physical exercise promoted improvement in the antioxidant apparatus and reduction of oxidative stress in the hepatic mitochondriae, exerting a protective effect.

In humans, the pathogenesis of DM is closely related to failure of pancreatic beta cells.^36,37^ In the present study, although the pathogenic trigger is different (induction by administration of Alloxan), it is important to note that physical training was able to attenuate the metabolic and morphological alterations caused by this disease. Our experimental model of DM was similar to type 1 DM that occurs in humans − a disease characterized by autoimmune destruction of pancreatic insulin-secreting beta cells.^37^ Although the benefits of physical training for treating type 1 DM in animal studies are well described in the literature,^38^ a recent clinical study reported the protective effect of physical exercise on the function of newly diagnosed human beta cell lines, with type 1 DM*.* ^37^ These findings reinforce the importance of physical training as a non-pharmacological intervention against DM. There are reports of individuals with DM type 1 who presented alterations in hepatic metabolism;^39^ however, further research is necessary with these patients to investigate mitochondrial alterations and the possible protective effect of physical exercise.

The results obtained in our work highlight some relevant aspects. One is the damage caused by DM to the hepatocytes and this is demonstrated by the ultrastructural analysis of the liver, which presented many alterations, such as reduced number of rough endoplasmic reticulum. This fact may indicate lower hepatic protein synthesis, increased mitochondrial diameter, mitochondrial hyperfunction and increased accumulated lipid droplets, which are usually associated with hepatic steatosis. Another important finding of our study is the influence of physical activity on the liver. This aspect was shown by the following factors: the increased protein/DNA ratio of the liver in the TC Group, recovery of hepatic glycogen and reduction in mitochondrial diameter in the TD Group. This study provides some evidence about the role of physical exercise as a therapeutic strategy against damage caused by DM to the liver.

## CONCLUSION

Experimental *diabetes mellitus* reduced the glycogen content and increased the mitochondrial diameter in the liver. The physical training protocol increased the protein/DNA ratio in the hepatic tissue in Control Group, recovered the hepatic glycogen content of the liver in Diabetic Group, as well as the mitochondrial diameter of this same group. Therefore, physical swimming training promotes important metabolic and morphological adaptations in diabetic animals.
